# Structural Barriers Associated with the Intersection of Traumatic Stress and Gun Violence: A Case Example of New Orleans

**DOI:** 10.3390/healthcare9121645

**Published:** 2021-11-27

**Authors:** Rahn Kennedy Bailey, Chikira H. Barker, Amit Grover

**Affiliations:** 1 Department of Psychiatry, Louisiana State University Health Sciences Center, New Orleans, LA 70112, USA; cbark1@lsuhsc.edu; 2 Department of Psychiatry, Charles R. Drew University of Medicine and Science, Los Angeles, CA 90059, USA; a_grover@kedren.org

**Keywords:** gun violence, COVID-19, Hurricane Katrina, African-Americans, traumatic stress

## Abstract

Gun violence drastically increased in urban cities following the ease of shutdown restrictions associated with the Coronavirus Pandemic. The association of gun violence and COVID-19 has highlighted the importance of taking a public health perspective, particularly as it relates to impacts on the Black community. In this article we discuss macro-level factors and community traumas in the city of New Orleans, an area that has had longstanding issues related to gun violence. Community structural issues, traumatic stress from disasters, and recommendations to address disparities in social determinants of health are discussed.

## 1. Structural Barriers Associated with the Intersection of Traumatic Stress and Gun Violence: A Case Example of New Orleans

The alarming increase in gun violence during the coronavirus pandemic has led to a shift in viewing gun violence in the greater context of a public health issue. The first reported case of the coronavirus in Louisiana was reported 9 March 2020, approximately two weeks after the conclusion of Mardi Gras celebrations in New Orleans. In the ensuing months, New Orleans gained national attention as one of the early hotspots of the coronavirus outbreak. While African-Americans represent under 60% of the population in New Orleans, they comprise over 72% of deaths due to COVID-19 [1]. In addition to the community trauma associated with grief and loss from death in the pandemic, the economic impact was devastating for residents as businesses closed and tourism declined. The coronavirus pandemic highlighted several concerns related to widespread community-level impacts: economic strain, education declines, limitations in social connections, and population physical and mental health.

**Figure 1:**
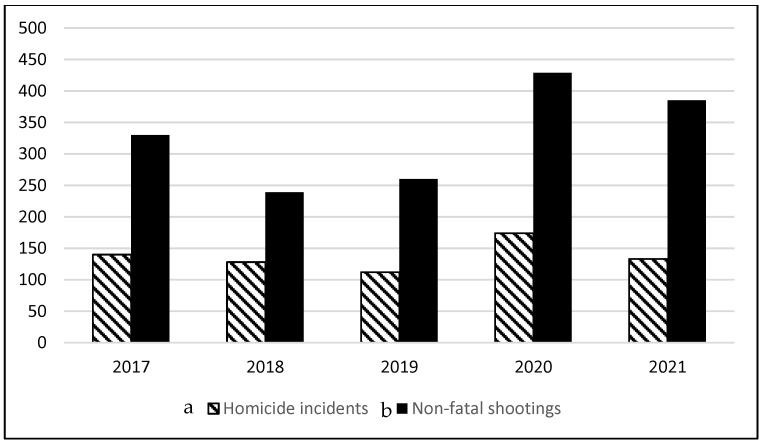
Homicide and non-fatal shooting incidents in New Orleans between 2017 and 2021^a,b^; ^a^ Homicide and non-fatal shooting incidents reported through September 2021; ^b^ Data collected from the city of New Orleans Office of Criminal Justice Coordination.

Figure 1 shows homicide and non-fatal shooting incidents in New Orleans from 2017 to the beginning of October of 2021. Year-to-date statistics from 2020 to 2021 indicated a 2.4% increase in homicide incidents with firearms, while non-fatal shootings increased by 37% [2]. This disturbing increase in gun violence post-shutdown was not isolated to New Orleans; many urban areas saw drastic increases in gun violence in the United States [3]. Given the clear resurgence of violent crime following lockdown, it has brought greater attention to the issue of gun violence from more of a public health framework, specifically in understanding the intersection of mental health and macro-level factors leading to gun violence.

### 1.1. Social Determinants of Health

Based on the concept of social justice and fairness, health equity is achieved when health disparities and structural social determinants that systematically contribute to those disparities for disadvantaged groups have been eliminated [4]. The city of New Orleans presents a unique example of examining gun violence from a social determinants of health (SDOH) framework. SDOH emphasize that a person’s neighborhood factors, economic instability, social or community connections, and access to quality education and health care has substantial impact on health [5].

The state of Louisiana has lower ratings for health-related outcomes, health factors, health behaviors, clinical care, and social/economic factors compared to the national average [6,7]. Within Louisiana, New Orleans ranks 32 out of 64 for county ranking health outcomes [6]. The public health factor of greatest concern for New Orleans has been violent crime disparities. Between 2008 and 2010, the rates for homicide far outpaced diabetes, cancer, kidney disease, and heart disease as a leading cause of death in Orleans Parish [8]. This disparity is very pronounced for African-American residents in New Orleans [8]. From a social determinants of health perspective, the reasons for more violent crime are complex. Community-level data has shown that urban areas and communities with greater income inequality (having simultaneous higher median income and higher levels of poverty) is associated with more gun violence. This suggests that gun violence is not specifically related to poverty but is likely to occur in areas where there is segregation based on race and economic disadvantage is greater [9].

Gun violence is not a new issue for New Orleans. Social determinants related to education, economic disparity, community disruption, and racial segregation have been ongoing areas of concern that may be associated with gun violence. Many of the problems have been exacerbated by the city being hit by Hurricane Katrina (29 August 2005), and more recently Hurricane Ida (29 August 2021). Both hurricanes devastated significant parts of the city, but the greatest damage was sustained in predominantly black communities in Hurricane Katrina [10]. The population of the Greater New Orleans area dropped by approximately 197,000 people following Katrina, with 64% of the people who were displaced and did not return to the city being African-American [10,11]. Rent drastically increased and over 70% of the units sustaining damage were primarily designated for low-income families [10].

Hurricane Katrina was an example of injustice and racism in all respects of response, recovery, and repair. It is possible that Hurricane Ida may produce the same impairment. While most residents were optimistic about the future of the city of New Orleans, a study found that residents reported that most of the recovery effort targeted those that were wealthy and white [12]. Ten years after Hurricane Katrina, African-American residents were more likely to report experiencing financial stressors, employment concerns, recovery issues, and having limited access to neighborhood amenities (grocery stores, restaurants, places for children to play) compared to white residents [13]. While all New Orleans residents believed that crime needed to be a city-wide priority, African-American residents reported feeling more vulnerable to violent crime and believed there was a lack of police presence in their neighborhoods [13].

**Figure 2:**
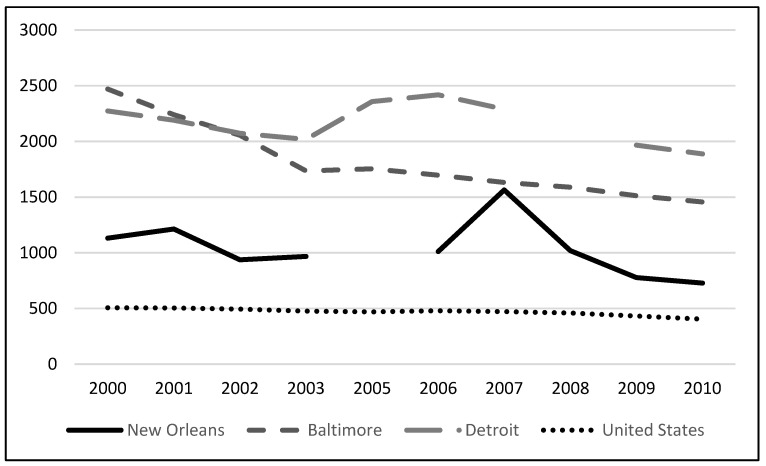
Rates of violent crime (per 100,000) for New Orleans, Baltimore, and Detroit. 2005 rates for New Orleans were not included in the Uniform Crime Report.

**Figure 3:**
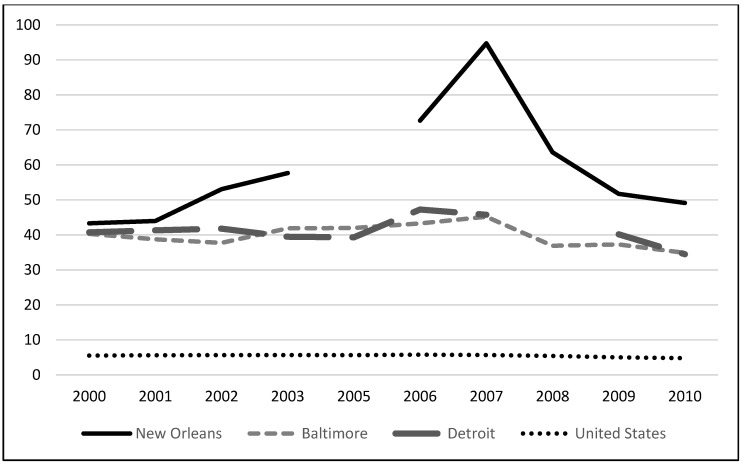
Murder/Manslaughter rates (per 100,000) for New Orleans, Baltimore, and Detroit. 2005 rates for New Orleans were not included in the Uniform Crime Report. 2006 rate calculations were adjusted to exclude displaced residents not currently residing in the city of New Orleans.

Violent crime and murder/manslaughter rates for New Orleans and other major U.S. cities in the years preceding and following Hurricane Katrina are listed in Figure 2 and Figure 3 [14]. While overall violent crime in New Orleans was lower compared to Detroit and Baltimore, the murder and homicide rates were higher. Rates spiked in 2006 and again in 2007 in the years immediately following Hurricane Katrina as the city worked through recovery.

### 1.2. Psychological Stress and Trauma: Hurricane Katrina and COVID Pandemic

Research has shown that when an individual is directly exposed to a pandemic/disaster they have a 30–40% chance of developing post-traumatic stress disorder [15]. This is concerning as African-Americans already have higher than normal rates of PTSD [16]. When compared to past infectious outbreaks/natural disasters we see similar results. Those who faced the Ebola epidemic in West Africa saw increased rates of PTSD and depressive symptoms [17]. Post-disaster research following Hurricane Katrina found that half of New Orleans residents had an anxiety-based mood disorder, with 30% meeting diagnostic criteria for post-traumatic stress disorder [18]. Death of family and friends was the strongest predictor of increased psychological distress among African-American survivors of Hurricane Katrina [19]. Given the recent experience of Hurricane Ida on the anniversary of Hurricane Katrina, we may expect a higher risk of experiencing psychological distress for those in the greater New Orleans area.

Results have been mixed regarding depression symptomology. Post-Katrina, overall rates in New Orleans for depressive episodes and substance use disorders were comparable to national rates [20]. While African-American evacuees had similar levels of depression as White Americans, African-Americans experienced higher levels of displacement stress [21]. Ali and colleagues [22] found that post-Katrina, African-Americans had 86% higher odds of screening positive for depression compared to White Americans; however, this disparity was largely eliminated once accounting for lower education level, social support, and previous hurricane-related traumatic experiences. This study further highlights the importance of considering how social determinants affects psychological symptoms.

While overall suicide rates decreased in 2020 during the initial phase of the coronavirus outbreak, there was a notable increase in reported depressive and anxiety symptoms. In one survey, 40% of respondents experienced depressive, anxiety, or pandemic-related traumatic stress in June of 2020 [23]. Psychological distress and suicidal ideation was significantly higher for African-American respondents, with African-American respondents also reporting the highest substance use in response to stress experienced during the pandemic [23]. African-Americans were two times more likely to have a friend/family member who had either died or had been hospitalized due to COVID-19 [24]. Therefore, higher rates of acquiring the virus and disproportionate hospitalizations and deaths in the Black community adds to increased symptoms of anxiety.

Despite higher rates of reported stress, substance use, and suicidal ideation during the COVID-19 pandemic, some initial studies report that African-Americans reported similar or lower levels of depression compared to Whites and other ethnic groups [25]. This suggests that other psychiatric disorders, such as anxiety, may be a significant drive of psychiatric disturbance. Many studies in the literature support anxiety disorders as being heavily associated with contemplation of suicide [26,27]. Suicide attempts in African-Americans was associated with a six-fold risk if the person had an anxiety disorder [28]. Perceived discrimination is associated with more anxiety symptoms, depressive symptoms, and suicidal ideation in African-Americans [29]. These examples highlight that African-Americans are particularly vulnerable to the negative mental health consequences of a national crisis.

### 1.3. Addressing Structural Inequalities: Gun Violence as a Public Health Issue

We acknowledge that gun violence is a complex, multifaceted issue with no simple solutions. Despite this fact, there are some very simple, salient issues that can be discussed and addressed as they pertain to gun violence: (1) The presence of guns is a known contributor; (2) Gun violence is a public health issue that warrants public health professionals being involved in solutions; (3) More research is needed pertaining to guns and gun violence; (4) Reducing structural barriers is needed to improve access to care; and (5) The dire need for interventions to address social determinants and anxiety symptoms to prevent gun violence.

### 1.4. Presence of Guns

At a minimum, we as a society need to acknowledge that easy access to guns is the primary problem. The ability to access guns, especially very powerful guns, has become a polarizing issue in which politics and financial gain have trumped public health concerns. Historically, White Americans have been the primary advocates for gun rights. This is a paradox given that middle-aged white men are one of the groups at most risk for suicide [30]. During a time in which COVID-19 has increased the psychological distress experienced by individuals and families, background checks for licensed gun sales have increased by 80% over the last year [31]. The greatest rate of gun ownership increase was seen in African-Americans, increasing by 58.2% the first half of 2020 compared to the same period in 2019 [32]. While this increase may be associated with national unrest from racial tensions and police injustice, this is particularly concerning as African-Americans are reporting significantly higher levels of psychological distress and substance use during the pandemic. Paired with cumulative trauma, depressive symptoms, agitation, and frustrations from macro-level inequities, greater access to deadly weapons can lead to more interpersonal and self-inflicted violence.

### 1.5. Gun Violence as a Public Health Issue

Gun violence must be viewed within the lens of community public health. Mental health, medical, and other public health professionals should be included in discussions with policymakers and law enforcement on addressing gun violence. Public health officials need to explain the idiosyncrasies associated with gun violence: punitive methods alone are not deterring gun violence. Mayor LaToya Cantrell with the city of New Orleans developed a task force in 2019 seeking to take a public health approach to addressing gun violence. Mental health professionals, academic faculty from local universities, law enforcement officials, city officials, and community non-profit organizations were included on the city’s task force. The Coronavirus outbreak occurred shortly after launching the task force. Given the community stress, health, and economic challenges associated with COVID-19, public health officials will be vital in bringing attention to how psychological stress and social inequities will be associated with increased gun violence in an already stressed system.

### 1.6. Structural Barriers to Research

Central to taking a total health approach to addressing gun violence is improving our research. Not only do we need more research, but we also need to make use of technology that has already been developed. Structural roadblocks, such as The Dickey Amendment, are preventing us from conducting more research on gun violence and preventative measures to reduce gun use [33]. The Dickey Amendment is a Congressional provision that has limited the use of federal funding for research or any measure promoting gun control. This emerged in response to research supporting the need for gun control and the Center of Disease Control’s subsequent actions to treat gun violence as a public health problem twenty years ago. Pressured from the National Rifle Association (NRA), congressional members passed legislation prohibiting research funding for studies that could be seen as advocating for gun control [34]. Despite efforts by President Obama to implement policies and encourage the CDC to conduct research related to gun violence, there has not been enough Congressional support to overturn the ban. Providing funding for more data-driven research is critical in addressing gun violence, particularly as it relates to potential contributors of community-level traumas and impacts from social health determinants.

### 1.7. Barriers to Accessing Care

A key issue in improving rates of gun violence is reducing the structural barriers preventing access to individual treatment for psychiatric concerns. Empirical data show that of those with a psychiatric condition only 31% of African Americans receive treatment versus 48% of White Americans [35]. A key component determining access to healthcare is geographical location [36], so making clinics more accessible is one method of reducing structural barriers for care. The increase in funding for Federally Qualified Health Centers (FQHCs) and school-based health clinics under the Affordable Care Act led to a growth in clinics; however, more oversight is needed to ensure that these clinics are being built in areas where the residents tend to be the most disadvantaged [37]. These clinics can increase access to behavioral health services in some of the most vulnerable communities. They can also reduce financial barriers such as lack of insurance or money for co-payments. Common reasons seen for poor access was low levels of insurance coverage and self-reported difficulty paying for medications/co-pays [36,38,39]. Other studies have shown that even a modest co-pay can affect medication adherence of low-income marginalized communities [40,41,42]. Those that are less fortunate will not present for regular appointments or be able to obtain their essential medications. This process will hinder their ability to receive the necessary care to prevent complications.

### 1.8. Need for Community-Level Interventions

Other barriers to mental health services in the highest-need communities is a lack of providers that are willing to provide service to the area. We should incentivize providers to select African-American neighborhoods. This can be achieved by forms of tax exemptions, subsidies, government funding, and local financial incentives. Many mental health providers are less likely to participate in public insurance programs (e.g., Medicare and Medicaid), which have lower reimbursement rates than commercial insurance [43]. In some states, psychiatrists are reimbursed less than primary care physicians even when billing for the same service [44]. Initial success has been demonstrated in increasing behavioral health services and cost-savings using alternative payment models, which provide financial incentives for integrating behavioral health into primary care settings, known as the collaborative care models [45].

Some members of minority communities are not informed about the potential benefits of preventative healthcare. Moreover, individuals might not be knowledgeable about the risks and complications of not utilizing such services. Therefore, education/promotion can be one of the vital components to ensure that healthcare is utilized optimally. This is important for forgotten communities. Making ethnic communities aware of the availability of certain health services that others can access routinely can facilitate patients to seek out these preventative services. A prime example of this is the initiative called Healthy People, launched by the Office of Disease Prevention and Health Promotion in 2010 [39]. One of the key objectives of this program is to “increase social marketing in health promotion and disease prevention” [46]. The CDC supports this process of social marketing as a key framework to spread health education to communities [36]. Customized health messages have shown to have better efficacy over generic, broad, and non-specific messaging [36]. Other health promotion campaigns are using barbershops and hair salons as potential opportunities for intervention. While some evaluation studies for education on hypertension and diabetes show promising results, more research is needed to evaluate the effectiveness of utilizing these locations for community-level interventions [47]. Specific interventions and studies are needed to address community-level education on trauma and anxiety-related symptoms to improve recognition and referrals for care in Black communities.

In addressing many of these structural barriers to improving access to mental health services and reducing gun violence, public health officials must acknowledge the longstanding distrust that African-Americans have due to mistreatment, bias, and discrimination. The cumulative effects of this historical victimization by medical, law enforcement, and government representatives likely leads to more anxiety and reduces willingness to seek help from the agencies that are supposed to serve this population.

## 2. Conclusions

Natural disasters, such as Hurricane Katrina, Hurricane Ida, and the Coronavirus Pandemic, are community traumas that have further exacerbated ongoing issues related to stress and economic disparities in a city already facing high levels of gun violence. For Black Americans, the experiences of cumulative trauma, racism, and chronic life stressors warrant further attention to the role that anxiety symptoms and related mood disorders may play in exacerbating the problem with gun violence. A population that may be experiencing these issues as a result of systemic failures leading to limited economic opportunities, educational challenges, and a competition for limited resources may resort to resolving conflict impulsively. It is the perfect storm for injury, particularly when firearms are readily available.

## Data Availability

The data presented in this study are available on request from the corresponding author.
